# Long-Term Effects of the Treatment of Depressive Female Inpatients in a Naturalistic Study: Is Early Improvement a Valid Predictor of Outcome?

**DOI:** 10.1155/2014/780237

**Published:** 2014-06-30

**Authors:** Elian Zuercher-Huerlimann, Martin grosse Holtforth, Ernst Hermann

**Affiliations:** ^1^Department of Psychology, University of Zurich, Binzmuehlestraße 14/19, 8050 Zurich, Switzerland; ^2^Department of Psychology, University of Bern, Fabrikstraße 8, 3012 Bern, Switzerland; ^3^Department of Psychology, University of Basel, Missionsstraße 60/62, 4055 Basel, Switzerland

## Abstract

*Objectives.* To examine the predictive value of early improvement for short- and long-term outcome in the treatment of depressive female inpatients and to explore the influence of comorbid disorders (CD). *Methods.* Archival data of a naturalistic sample of 277 female inpatients diagnosed with a depressive disorder was analyzed assessing the BDI at baseline, after 20 days and 30 days, posttreatment, and after 3 to 6 months at follow-up. Early improvement, defined as a decrease in the BDI score of at least 30% after 20 and after 30 days, and CD were analyzed using binary logistic regression. *Results.* Both early improvement definitions were predictive of remission at posttreatment. Early improvement after 30 days showed a sustained treatment effect in the follow-up phase, whereas early improvement after 20 days failed to show a persistent effect regarding remission at follow-up. CD were not significantly related neither at posttreatment nor at follow-up. At no time point CD moderated the prediction by early improvement. *Conclusions.* We show that early improvement is a valid predictor for short-term remission and at follow-up in an inpatient setting. CD did not predict outcome. Further studies are needed to identify patient subgroups amenable to more tailored treatments.

## 1. Introduction

Despite ongoing developments in the pharmacological and psychotherapeutic treatment of depressive disorders, recurrent depressive illness remains one of the predominant causes of disability worldwide [1, 2]. Therefore, it is necessary to identify predictors of therapeutic outcomes to further improve the development of effective treatments. In treatment research, there is a wide agreement that the combination of pharmacotherapy and psychotherapy is more promising with respect to achieving remission and preventing relapse than either used alone [3–5]. Similarly, recent meta-analyses have focused on the benefits of psychotherapy in addition to pharmacotherapy alone and associate them with a higher probability of remission and a lower risk of relapse [4, 6].

Concerning the role of pharmacotherapy, findings regarding the onset of the therapeutic effects of antidepressants have been heterogeneous. Several meta-analyses showed that drug responses may occur within the first two weeks [7–9]. However, a current meta-analysis by Iovieno and Papakostas [10] shows a high heterogeneity in the relative efficacy of pharmacotherapy in comparison to placebo in several studies, and findings by Tedeschini et al. [11] suggest that a minimum of four weeks is needed to reliably detect drug versus placebo effects. Concerning the role of psychotherapy, early gains have been identified as important predictors of response and positive outcomes [12–14], as well as of stable response and remission in the combined depression treatment of outpatients in RCT designs [15–17].

Inpatients represent a substantial portion of the population of depressed patients and act as the biggest driver of costs in the treatment of depression [18]. The question arises as to whether these previous findings can be generalized to inpatient settings. It is reasonable to assume that patients requiring inpatient care most likely suffer from more severe depressive episodes [19]. Furthermore, the occurrence of comorbid mental disorders has been found to be more prevalent among more severely depressed patients [2]. As a consequence, a higher number of confounding variables including comorbid mental disorders should be considered when investigating depressed inpatients.

Considering the high relapse rates for formerly depressed patients [2], it is of particular importance to further examine the sustainability of treatment effects after discharge. The predictive value of early improvement has been studied in a large naturalistic study of inpatients with major depression fairly recently [20]. Yet, little is known about the sustainability of treatment effects and the predictive value of early improvement over an extended period of time. To our knowledge, similar analyses of the influence of comorbid disorders do not yet exist. Hence, the aim of this study was to analyze the predictive value of early improvements on long-term effects in a combined therapy for depressed female patients in a naturalistic inpatient setting, taking comorbid disorders into account. Compared to men, women have a twofold higher risk for the development of a depressive disorder [21]; their risk for disease recurrence is higher, and the duration of their disease episodes is longer [2]. For all of these reasons, women in particular represent a subpopulation worth researching for specific outcome predictors.

## 2. Methods

### 2.1. Sample and Treatment

**Figure 1:**
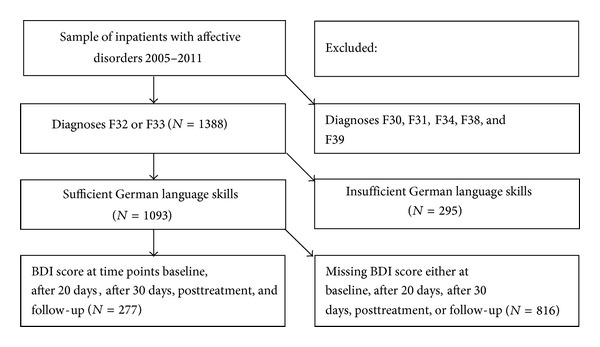
Flow chart: recruitment of patients.

The current data were collected in a private psychiatric hospital for women in Switzerland. Whereas the clinic is privately run, it serves the community, regardless of the patients' type of insurance. All patients meeting the diagnostic criteria for either a depressive episode (F32) or a recurrent depressive disorder (F33) as first or second diagnosis according to the ICD-10 [22] who were being treated during the years 2005–2011 were eligible for inclusion into the current analysis. Excluded were all patients with other affective disorders, such as manic episodes (F30); bipolar disorders (F31); and persistent (F34), other (F38), or unspecified mood disorders (F39), and patients with insufficient German language skills (see Figure 1). For description of comorbid disorders, persistent mood disorders (F34) were included (see Table 2). Sociodemographic variables (age, family status, education, and working status) were collected through routine intake documentation. During the course of treatment, this documentation was supplemented with diagnostic and clinical data, such as disease specifiers (single versus recurrent), comorbid disorders, and the number of previous treatments.

Individual psychotherapies consisted of 3 to 4 sessions of 50 minutes per week using cognitive-behavioral treatment (CBT) interventions for depression [23] throughout the whole stay. When indicated, significant others and relevant persons from the professional environment were involved. Finally, strategies for the maintenance of therapeutic gains and relapse prevention were developed. In addition to CBT, all patients participated in a standard group therapy program consisting of assertiveness training, progressive muscle relaxation, walking and movement therapy, and art and occupational therapy. Almost all patients were also treated with psychoactive medication (antidepressants, neuroleptics, tranquilizer, or mood stabilizers) and if indicated, they also received medication treatment for somatic disorders.  Table 1 gives an overview of the type of medication taken at baseline and posttreatment. A clear majority was treated with antidepressants, and this proportion increased at the posttreatment stage. Additionally, the use of neuroleptics and mood stabilizers increased. However, the prescription of tranquilizers decreased considerably during treatment. The same changes were found regarding combinations of an antidepressant with any other medication category. In total, eight of the 277 patients did not receive any medication, neither at baseline nor at posttreatment. Due to the vast number of possible combinations, we did not include this information in the analyses.

### 2.2. Data Collection and Definitions

The archival data used were collected using a naturalistic design. The data were archived anonymously and did not allow for any personal identification. Feedback on the progress of symptoms was supplied for the treated patients. As data collection was conducted before and independent of this analysis, there was no interference with the aim of the present investigation of outcome predictors. The research was conducted in accordance with the American Psychological Association's ethical principles [24] and in compliance with precepts of the Declaration of Helsinki [25].

Subjects rated the severity of their depressive symptoms using the Beck Depression Inventory (BDI) [26] at the beginning and at the end of the treatment, as well as every ten days during treatment.* Remission* at the time points posttreatment and at follow-up was defined as measuring a BDI score of 11 or less [26]. This study focuses on assessments at baseline, after 20 days, after 30 days after treatment, and throughout follow-up (three to six months after hospitalization; *M* = 133.68, SD = 25.76 days). Patients with missing values for any of the five assessments were excluded (see Figure 1). Owing to the investigation of treatment sustainability at follow-up, the loss of a considerable number of patients to attrition (*N* = 813) was accepted.

*Early improvement *was defined as a decrease in the BDI score of at least 30%, compared to the baseline value, according to a naturalistic study by Henkel et al. [20]. According to the literature, there are different time points from 2 up to 8 weeks after baseline when early improvement is measured [15–17, 20]. We have decided to conduct our analyses for two different time points: after 20 days and after 30 days after baseline assessment. The latter considers the required minimum of four weeks of medication use, which is intended to minimize the contribution of placebo effects [11]. The early improvement variable was coded dichotomously, dividing the sample into two subgroups: nonearly improvers and early improvers, reported in the following as EI20/NEI20 (*n* = 160; *n* = 117) and EI30/NEI30 (*n* = 176; *n* = 101) for measurements after 20 and 30 days of treatment, respectively.

To investigate the influence of comorbid disorders, the variable* comorbid disorders* with two dichotomous values* (CD/NCD)* was defined: no comorbid disorder (NCD) (*n* = 95), and any comorbid disorder(s) (CD) (*n* = 182). The types and combinations of one or more present comorbid disorder in our sample cover an extensive range of ICD-10 diagnosis for mental and behavioral disorders. Because of this heterogeneity a further categorization of the type or number of comorbid disorders was not conceivable in this study. The descriptive data of all comorbid disorders can be found in Table 2.

### 2.3. Statistical Analyses

All statistical calculations were performed using the PASW Version 18 for Windows. To describe the two study groups with and without early improvement and to investigate differences in demographic and clinical characteristics, two-tailed unpaired *t*-tests and *χ* ^2^-tests were computed. Binary logistic regression analyses were computed to examine the association of the variables early improvement and comorbid disorders with remission or nonremission at posttreatment and at follow-up, controlling for the variables baseline BDI, age, and treatment duration.

## 3. Results

### 3.1. Description of the Sample

The sample consisted of *N* = 277 patients, whose demographics and clinical characteristics are described in Table 3 for the subgroups EI30 and NEI30. Because results did not differ for the subgroups EI20 and NEI20 we did not report this data separately. For lack of space, we also chose to focus in the text on the early improvement definition made 30 days after baseline (EI30/NEI30), whereas results for EI30/NEI30 are given in the tables.

Table 3 reports the sample demographics. Of demographic variables, only average age differed significantly between EI30 und NEI30. Whereas the occurrence of a single or recurrent depressive episode showed no significant differences in the subgroups, comorbid disorders were diagnosed for about two-thirds of the patients, and differed in distribution between EI30 and NEI30. Regarding previous in- or outpatient treatments, no significant differences between the subgroups were found. However, treatment duration was significantly longer for patients in the NEI30 subgroup.

**Figure 2:**
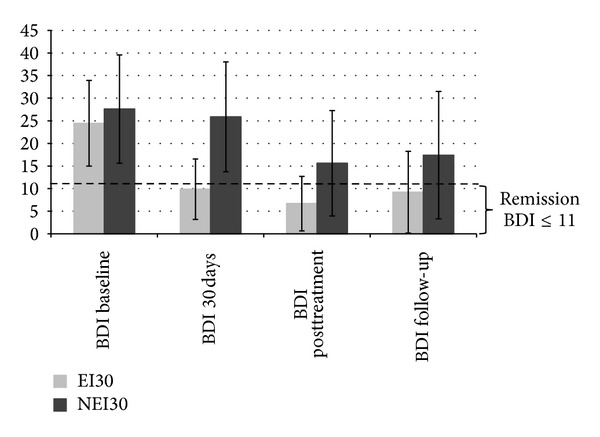
BDI scores overtime by study groups EI30 and NEI30.

Table 4 reports BDI scores at baseline, 30 days, posttreatment, and follow-up. These BDI scores were used as the base of further analyses using binary logistic regression. As can be seen in Figure 2 displaying the change of the BDI scores for the subgroups EI30 and NEI30 over time, the BDI scores of the EI30 group reached the level of remission status after 30 days (≤11 points) and remained below the threshold throughout the observation time.

### 3.2. Posttreatment Outcomes

The variables baseline BDI, age, and treatment duration differed significantly between the subgroups EI20 and NEI20, as well as between EI30 and NEI30 (Table 3), so that we included these variables in the binary logistic regression as covariates. Whereas for early improvement at 30 days, baseline BDI and treatment duration showed a significant effect, age did not. After controlling for these covariates, the factor EI30/NEI30 showed a significant effect on remission at posttreatment (Table 6) showing that patients with early improvement had a higher probability to remit. However, neither comorbid disorders (CD/NCD) nor their interaction with early improvement (EI30/NEI30) predicted remission at posttreatment. The regression analysis with EI20/NEI20 displays similar results as the EI30/NEI30 calculations (Table 5).

### 3.3. Follow-Up Outcomes

Analysis of EI30/NEI30 showed a significant effect of baseline BDI on remission, whereas treatment duration and age did not. After controlling for these covariates, the factor EI30/NEI30 showed a significant effect on remission at follow-up. Patients with early improvement had a higher probability of remission. The factor comorbid disorders (CD/NCD) and its interaction with early improvement (EI30/NEI30) did not have any significant effect regarding remission at follow-up (Table 6). For early improvement after 20 days, binary logistic regression yielded little differences in results compared to the improvement after 30 days. However, the early improvement (EI20/NEI20) is not predicting remission beyond posttreatment (Table 5). In addition, treatment duration had a significant effect on remission.

## 4. Discussion

Recently, several studies and meta-analyses have addressed the predictive value of an early improvement pattern in combination with treatment of CBT and antidepressant medication. However, most of these analyses were conducted in an outpatient setting and did not extend to a time horizon beyond 10 weeks. This study investigates whether early improvement is also a valid predictor for treatment response and long-term sustainability of combined psychotherapy and pharmacotherapy in depressed female subjects in an inpatient clinic. Commonly, an inpatient population presents a range from mild to severe symptoms of depression.

After controlling for baseline severity of depressive symptoms, age, and treatment duration, patients with early improvement had a higher probability to show remission at posttreatment. Early improvement was predictive for remission independent of any comorbid disorders. Patients with or without comorbid disorders (CD/NCD) did not differ in their treatment outcomes regarding remission, nor did CD/NCD interact with early improvement in the prediction of outcomes. These findings occurred in both early improvement after 20 days and after 30 days of treatment. At posttreatment both early improvement definitions had the same explanatory power concerning the prediction of remission.

The overall depression mean showed a manifest improvement as compared to baseline three to six months after termination of inpatient treatment (see Figure 2 and Table 4). However, mean BDI scores increased from posttreatment to follow-up by 2.25 BDI points so that the average BDI score over all patients cannot be considered as remission of the total group anymore (Table 4). Nevertheless, the distinction between early improvers and nonearly improvers provides interesting information. Patients who show early improvement after 30 days of treatment have a significantly higher probability to still be a “remitter” at follow-up. This prediction can only be made based on the early improvement assessment after 30 days, whereas the early improvement after 20 days does not offer this predictive information. Comorbidity during follow-up neither has a significant predictive value nor is it indicative of a detectable interaction with early improvement.

Our findings on significant outcome predictors are in accordance with comparable findings in RCTs with outpatient samples [15–17] and in a naturalistic study with inpatients [20]. The definition of early improvement as a decrease of 30% in BDI score compared to baseline for the two measurement times after 20 days and after 30 days of treatment does not provide any differential prediction for remission at posttreatment. But for remission at follow-up the assessment after 30 days offers predictive information, whereas the measurement after 20 does not. Furthermore, placebo effects of pharmacotherapy as a potential explanation for early symptom reduction are minimized [11]. In a long-term perspective, measuring early improvement after 30 days provides the advantage of a more reliable prediction. However, future research will need to define a consensual maximum time limit for labeling improvements as “early.”

In general, attention should be paid to the aggravation of the self-reported depressive symptoms from posttreatment to follow-up, represented in a reincrease of the BDI scores after termination of inpatient treatment (Table 4). Obviously, not all patients achieve a satisfying transfer of in-therapy gains into their customary environments. This observation might be explained by the relative stability of the social network in comparison to the patient's psychological functioning, which most likely has undergone substantial changes during treatment, as long as it was not integrated into the inpatient treatment. In the inpatient setting, moreover, the relational environment is considered a particularly important factor with high therapeutic potential; the ward has its own social environment, including other inpatients and health-care professionals, in addition to specific group therapies and activities [27]. As a consequence, it seems reasonable to expect a destabilization of the patient's well-being, once having to do without this support and being back in their largely unchanged social network [28]. Consequently, when investigating the symptom trajectories of former inpatients over longer periods, these are factors to be incorporated into the investigation.

Neither the existence of comorbid disorders at baseline nor related interaction with early improvement significantly predicted remission. These findings were in contrast to our expectations, given the fact that comorbid disorders had previously been found to be associated with a greater severity of symptoms and lower treatment response rates [2]. However, the descriptives in Table 4 show that patients with CD and without early improvement evidently display higher BDI scores than the other patients. Consequently, it may be assumed that the patients with CD present with the most heterogeneous and complex problem pattern. As a consequence further research may better capture the complexity of these patients' problems at intake.

Our study has several limitations. It is based on naturalistic inpatient data, and thus several factors could not be controlled for, such as a further distinction of comorbid disorders, their treatment during the stay, or any treatments thereafter. Additionally, due to the power restrictions, the differential effects of various antidepressant medications and combinations with other psychotropic drugs could not be analyzed in detail. Nevertheless, it seems worth mentioning that the prescription of antidepressants, neuroleptics, and mood stabilizers increased from baseline to posttreatment, whereas the use of tranquilizers decreased. This scheme represents common clinical practice in the time course of a state-of-the-art treatment of depressive patients. Whereas our findings for female inpatients are not generalizable to males, they do have a significant clinical relevance, given that females account for two-thirds of all depressed patients [21].

## 5. Conclusion

In summary, we replicated the previous findings on the predictive value of early improvement for outcomes at treatment termination in a naturalistic inpatient setting. In addition, we can confirm the predictive value of early improvement also in a long-term perspective. We showed that early improvement is associated with remission at the end of the hospitalization and at follow-up three to six months thereafter. The diagnosis of comorbid disorders did not allow for better outcome predictions, which is probably due to the heterogeneity of this group. Future research may assess the treatment of comorbid mental disorders, as well as the recovery process in greater detail, accounting for other factors, such as treatment methods, the therapeutic relationship, patient expectations, and therapist influences [29]. Furthermore, a closer investigation of gender-specific factors in inpatient treatment and the transfer of the treatment benefits into daily life in the customary environment may also promise further advances in the care of depressed inpatients.

## Figures and Tables

**Table 1:** Frequencies and changes in medications used over the course of treatment.

Medication type	Baseline	Posttreatment	Change
Antidepressants	73.6% (*n* = 204)	82.3% (*n* = 228)	8.7% (*n* = 24)
Neuroleptics	22.4% (*n* = 62)	35.7% (*n* = 99)	13.3% (*n* = 37)
Tranquilizer	26.4% (*n* = 73)	7.9% (*n* = 22)	−18.5% (*n* = 51)
Mood stabilizer	8.7% (*n* = 24)	14.8% (*n* = 41)	6.11% (*n* = 17)
Antidepressants + neuroleptics	25.5% (*n* = 52)	39.0% (*n* = 89)	13.5% (*n* = 37)
Antidepressants + tranquilizer	27.0% (*n* = 55)	9.6% (*n* = 22)	−17.4% (*n* = 33)
Antidepressants + mood stabilizer	10.3% (*n* = 21)	14.5% (*n* = 33)	4.2% (*n* = 12)

**Table 2:** Types and frequencies of comorbid disorders (ICD-10).

Type of comorbid disorder	1st CD	2nd CD	3 and more CDs
	(*n* = 182)	(*n* = 87)	(*n* = 41)
F0 organic, including symptomatic, mental disorders	2	0	0
F1 mental and behavioural disorders due to psychoactive substance use	18	12	15
F2 schizophrenia, schizotypal and delusional disorders	5	1	0
F34.1 dysthymic disorder	11	1	1
F4 neurotic, stress-related, and somatoform disorders	66	37	7
F5 behavioural syndromes associated with physiological disturbances and physical factors	16	10	2
F6 disorders of adult personality and behaviour	50	12	6
F7 mental retardation	1	1	1
F8 disorders of psychological development	0	0	0
F9 behavioural and emotional disorders with onset usually occurring in childhood and adolescence	5	6	6
Z factors influencing health status and contact with health services	8	7	3

**Table 3:** Demographic and clinical characteristics of subjects by study groups.

Characteristics	Group with early improvement (EI30)	Group without early improvement (NEI30)	*χ* ^2^/*t*	df	*P* value
	*N* = 176	*N* = 101			
Average age (years)	45.50 (SD = 16.72)	39.93 (SD = 14.16)	−2.817	275	.005
Family status					
Single	25.6% (*n* = 45)	26.7% (*n* = 27)	.979	2	.614
Married/with partner	59.7% (*n* = 105)	54.5% (*n* = 55)			
Divorced/widowed	14.8% (*n* = 26)	18.8% (*n* = 19)			
Education					
Primary and secondary school	75.6% (*n* = 133)	83.2% (*n* = 84)	2.433	2	.295
Upper secondary school (matura)	14.8% (*n* = 26)	8.9% (*n* = 9)			
University or other tertiary education	9.7% (*n* = 17)	7.9% (*n* = 8)			
Working status					
Full-time/part-time	36.4% (*n* = 64)	41.6% (*n* = 42)	1.787	3	.650
Unemployed	60.2% (*n* = 106)	56.4% (*n* = 57)			
Student	2.8% (*n* = 5)	1.0% (*n* = 1)			
Retired	0.6% (*n* = 1)	1.0% (*n* = 1)			
Depressive disorder					
First episode	25.6% (*n* = 45)	23.8% (*n* = 24)	.112	1	.775
Recurrent episode	74.4% (*n* = 131)	76.2% (*n* = 77)			
Comorbid disorder					
No	43.2% (*n* = 76)	18.8% (*n* = 19)	16.913	1	.000
Yes	56.8% (*n* = 100)	81.2% (*n* = 82)			
Number of inpatient treatments	1.17 (SD = 1.72)	1.37 (SD = 1.74)	.910	275	.364
Number of outpatient treatments	1.09 (SD = .987)	1.29 (SD = 1.08)	1.538	275	.125
Treatment duration	85.88 (SD = 36.41)	114.50 (SD = 39.49)	6.106	275	.000

**Table 4:** Descriptives by study group and time of measurement.

Descriptives by study groups		Baseline BDI mean (SD)	30 days BDI mean (SD)	Posttreatment BDI mean (SD)	Follow-up BDI mean (SD)
Early improvement	Comorbid disorders				
NEI30	NCD (*n* = 19)	25.63 (13.24)	23.47 (12.41)	9.42 (5.95)	9.89 (8.56)
	CD (*n* = 82)	28.04 (11.71)	26.44 (12.09)	17.04 (12.22)	19.13 (14.59)
	Total (*n* = 101)	**27.59 (11.98)**	**25.88 (12.15)**	**15.60 (11.67)**	**17.40 (14.10)**

EI30	NCD (*n* = 76)	23.82 (9.83)	9.04 (6.69)	6.12 (5.71)	7.84 (8.11)
	CD (*n* = 100)	24.93 (9.22)	10.51 (6.62)	7.12 (6.26)	10.23 (9.58)
	Total (*n* = 176)	**24.45 (9.48)**	**9.87 (6.67)**	**6.69 (6.03)**	**9.20 (9.03)**

Total	NCD (*n* = 95)	24.18 (10.55)	11.93 (9.95)	6.78 (5.88)	8.25 (8.20)
	CD (*n* = 182)	26.34 (10.50)	17.69 (12.35)	11.59 (10.62)	14.24 (12.85)
	Total (*n* = 277)	**25.60 (10.55)**	**15.71 (11.89)**	**9.94 (9.53)**	**12.19 (11.80)**

**Table 5:** Binary logistic regression analyses of the covariates/factors associated with treatment outcome at posttreatment and follow-up for the early improvement definition after 20 days (EI20/NEI20).

	*B* (SE)	OR	95% CI	*P* value
Posttreatment				
Baseline BDI	−.11 (.02)	.89	.86–.93	.000
Treatment duration	−.01 (.01)	.99	.98–1.00	.016
Age	.00 (.01)	1.00	.98–1.02	.997
EI20/NEI20	−1.15 (.39)	.315	.15–.67	.003
CD/NCD	.18 (.46)	1.19	.48–2.95	.705
Interaction EI20/NEI20 × CD/NCD	.71 (.74)	2.04	.48–8.78	.337

Follow-up				
Baseline BDI	−.06 (.02)	.94	.92–.97	.000
Treatment duration	−.01 (.01)	1.00	.96–1.00	.048
Age	.01 (.01)	1.01	1.00–1.03	.142
EI20/NEI20	−.53 (.33)	.59	.31–1.13	.114
CD/NCD	.26 (.38)	1.30	.62–2.71	.493
Interaction EI20/NEI20 × CD/NCD	.46 (.63)	1.58	.46–5.40	.468

**Table 6:** Binary logistic regression analyses of the covariates/factors associated with treatment outcome at posttreatment and follow-up for the early improvement definition after 30 days (EI30/NEI30).

	*B* (SE)	OR	95% CI	*P* value
Posttreatment				
Baseline BDI	−.12 (.02)	.89	.86–.92	.000
Treatment duration	−.01 (.01)	.99	.98–1.00	.023
Age	−.00 (.01)	.99	.98–1.02	.913
EI30/NEI30	−1.29 (.39)	.28	.13–.59	.001
CD/NCD	.15 (.44)	1.16	.49–2.72	.739
Interaction EI30/NEI30 days × CD/NCD	.90 (.82)	2.45	.50–12.13	.272

Follow-up				
Baseline BDI	−.06 (.01)	.94	.92–.97	.000
Treatment duration	−.01 (.00)	.99	.99–1.00	.076
Age	.01 (.01)	1.01	1.00–1.03	.148
EI30/NEI30	−.92 (.34)	.40	.21–.78	.007
CD/NCD	.04 (.36)	1.04	.51–2.11	.909
Interaction EI30/NEI30 × CD/NCD	1.18 (.69)	3.25	8.34–12.67	.089

## References

[1] Mathers C, Fat DM, Boerma JT (2008). *The Global Burden of Disease*.

[2] Richards D (2011). Prevalence and clinical course of depression: a review. *Clinical Psychology Review*.

[3] Cuijpers P, Dekker J, Hollon SD, Andersson G (2009). Adding psychotherapy to pharmacotherapy in the treatment of depressive disorders in adults: a meta-analysis. *Journal of Clinical Psychiatry*.

[4] Guidi J, Fava GA, Fava M, Papakostas GI (2011). Efficacy of the sequential integration of psychotherapy and pharmacotherapy in major depressive disorder: a preliminary meta-analysis. *Psychological Medicine*.

[5] Dekker J, Van HL, Hendriksen M (2013). What is the best sequential treatment strategy in the treatment of depression? Adding pharmacotherapy to psychotherapy or vice versa?. *Psychotherapy and Psychosomatics*.

[6] Oestergaard S, Møldrup C (2011). Optimal duration of combined psychotherapy and pharmacotherapy for patients with moderate and severe depression: a meta-analysis. *Journal of Affective Disorders*.

[7] Lam RW (2012). Onset, time course and trajectories of improvement with antidepressants. *European Neuropsychopharmacology*.

[8] Papakostas GI, Perlis RH, Scalia MJ, Petersen TJ, Fava M (2006). A meta-analysis of early sustained response rates between antidepressants and placebo for the treatment of major depressive disorder. *Journal of Clinical Psychopharmacology*.

[9] Taylor MJ, Freemantle N, Geddes JR, Bhagwagar Z (2006). Early onset of selective serotonin reuptake inhibitor antidepressant action: systematic review and meta-analysis. *Archives of General Psychiatry*.

[10] Iovieno N, Papakostas GI (2012). Correlation between different levels of placebo response rate and clinical trial outcome in major depressive disorder: a meta-analysis. *Journal of Clinical Psychiatry*.

[11] Tedeschini E, Fava M, Papakostas GI (2011). Placebo-controlled, antidepressant clinical trials cannot be shortened to less than 4 weeks’ duration: a pooled analysis of randomized clinical trials employing a diagnostic odds ratio-based approach. *The Journal of Clinical Psychiatry*.

[12] Busch AM, Kanter JW, Landes SJ, Kohlenberg RJ (2006). Sudden gains and outcome: a broader temporal analysis of cognitive therapy for depression. *Behavior Therapy*.

[13] Hunnicutt-Ferguson K, Hoxha D, Gollan J (2012). Exploring sudden gains in behavioral activation therapy for Major Depressive Disorder. *Behaviour Research and Therapy*.

[14] Kelly MAR, Roberts JE, Ciesla JA (2005). Sudden gains in cognitive behavioral treatment for depression: when do they occur and do they matter?. *Behaviour Research and Therapy*.

[15] Tadić A, Helmreich I, Mergl R (2010). Early improvement is a predictor of treatment outcome in patients with mild major, minor or subsyndromal depression. *Journal of Affective Disorders*.

[16] Van HL, Schoevers RA, Kool S, Hendriksen M, Peen J, Dekker J (2008). Does early response predict outcome in psychotherapy and combined therapy for major depression?. *Journal of Affective Disorders*.

[17] van Calker D, Zobel I, Dykierek P (2009). Time course of response to antidepressants: predictive value of early improvement and effect of additional psychotherapy. *Journal of Affective Disorders*.

[18] Luppa M, Heinrich S, Angermeyer MC, König H, Riedel-Heller SG (2007). Cost-of-illness studies of depression. *Journal of Affective Disorders*.

[19] Kleine-Budde K, Müller R, Kawohl W, Bramesfeld A, Moock J, Rössler W (2013). The cost of depression—a cost analysis from a large database. *Journal of Affective Disorders*.

[20] Henkel V, Seemüller F, Obermeier M (2009). Does early improvement triggered by antidepressants predict response/remission?—Analysis of data from a naturalistic study on a large sample of inpatients with major depression. *Journal of Affective Disorders*.

[21] van de Velde S, Bracke P, Levecque K (2010). Gender differences in depression in 23 European countries. Cross-national variation in the gender gap in depression. *Social Science and Medicine*.

[22] World Health Organization (1993). *The ICD-10 Classification of Mental and Behavioural Disorders: Diagnostic Criteria for Research*.

[23] Hautzinger M (2003). *Kognitive Verhaltenstherapie bei Depressionen: Behandlungsanleitung und Materialien*.

[24] American Psychological Association Ethical principles of psychologists and code of conduct including 2010 amendments. http://www.apa.org/ethics/code/principles.pdf.

[25] World Medical Association Declaration of Helsinki—Ethical principles for research involving human subjects. http://www.wma.net/en/30publications/10policies/b3.

[26] Hautzinger M, Bailer M, Worall H, Keller F (1996). *Beck-depressions-inventar (BDI): Bearbeitung der deutschen Ausgabe. Testhandbuch*.

[27] Mattke D, Zeeck A, Strauß B, Strauß B, Mattke D (2012). Stationäre und teilstationäre Gruppenpsychotherapie. *Gruppenpsychotherapie*.

[28] Pinsof WM, Zinbarg RE, Lebow JL (2009). Laying the foundation for progress research in family, couple, and individual therapy: the development and psychometric features of the initial systemic therapy inventory of change. *Psychotherapy Research*.

[29] Lambert MJ, Lambert MJ (2013). The efficacy and effectiveness of psychotheray. *Bergin and Garfield's Handbook of Psychotherapy and Behavior Change*.

